# A Robust Predicted Performance Analysis Approach for Data-Driven Product Development in the Industrial Internet of Things

**DOI:** 10.3390/s18092871

**Published:** 2018-08-31

**Authors:** Hao Zheng, Yixiong Feng, Yicong Gao, Jianrong Tan

**Affiliations:** State Key Laboratory of Fluid Power and Mechatronic Systems, Zhejiang University, Hangzhou 310027, China; 11025064@zju.edu.cn (H.Z.); gaoyicong@zju.edu.cn (Y.G.); egi@zju.edu.cn (J.T.)

**Keywords:** Industrial Internet of Things, performance analysis, product development, support vector regression

## Abstract

Industrial Internet of Things (IoT) is a ubiquitous network integrating various sensing technologies and communication technologies to provide intelligent information processing and smart control abilities for the manufacturing enterprises. The aim of applying industrial IoT is to assist manufacturers manage and optimize the entire product manufacturing process to improve product quality and production efficiency. Data-driven product development is considered as one of the critical application scenarios of industrial IoT, which is used to acquire the satisfied and robust design solution according to customer demands. Performance analysis is an effective tool to identify whether the key performance have reached the requirements in data-driven product development. The existing performance analysis approaches mainly focus on the metamodel construction, however, the uncertainty and complexity in product development process are rarely considered. In response, this paper investigates a robust performance analysis approach in industrial IoT environment to help product developers forecast the performance parameters accurately. The service-oriented layered architecture of industrial IoT for product development is first described. Then a dimension reduction approach based on mutual information (MI) and outlier detection is proposed. A metamodel based on least squares support vector regression (LSSVR) is established to conduct performance prediction process. Furthermore, the predicted performance analysis method based on confidence interval estimation is developed to deal with the uncertainty to improve the robustness of the forecasting results. Finally, a case study is given to show the feasibility and effectiveness of the proposed approach.

## 1. Introduction

With the rapid integrated development of information technologies such as artificial intelligence, big data and Internet of Things in the manufacturing industries, industrial IoT is considered a crucial manufacturing infrastructure to efficiently change how the products are customized, manufactured and delivered [1,2]. Currently, industrial IoT is still in the preliminary stages with respect to development, deployment and application, and IoT-based solutions are key enabling technologies for implementing smart manufacturing and Industry 4.0 [3,4] .

In the applications of industrial IoT such as health management [5] and quality control [6], massive equipment- and human-generated data will create huge challenges to manage such data in order to improve the competitiveness of manufacturing enterprises. It is quite common that many manufacturers either do not store data or know little about how to apply these data [7,8]. Product development is a vital application scenario for implementing industrial IoT, which can directly determine the final quality and total lifecycle cost of products [9,10,11]. Data-driven product development can help designers enhance their organization’s competitive edge by uncovering business patterns, novel insights, and implicit knowledge. Traditional product development methods such as axiomatic design [12] and function-behavior-structure model [13,14] mainly focus on the summary of designers’ cognitive activities and introduce corresponding reasoning algorithms to solve design problems, while how to exploit the huge contextualized through-life data is least supported. There is clearly a gap in the availability of design support tools for such an important phase.

In general, the product development process usually involves the following fundamental activities: problem and requirements formulation, exploring alternative solutions, evaluation and documentation of the results [15]. By perceiving the collected product data such as users’ preference, design knowledge and maintenance records, a great deal of valuable information can be excavated to assist designers search the design space explicitly. In response, many scholars have focused considerable attention on data-driven product development and proposed lots of gratifying achievements. From the perspective of knowledge processing, Zha and Sriram presented a knowledge-intensive support paradigm for platform-based product development, and developed a prototype system which can realize design knowledge capture, representation and management [16]. Chu et al. put forward an elaborate expert system based on rough set theory and self-organizing maps, which can utilize the product design knowledge to guide engineers to reduce design space [17]. In the closed-loop product lifecycle management, diverse kinds of data are collected from each lifecycle phase, shared with other lifecycle phases and used for specific objectives. By transforming the data into useful knowledge and information, it is possible for engineers to improve product performance in the product development phase. From the perspective of data application, Shin proposed a design modification supporting approach for product improvement based on product usage data by using diverse data processing techniques [18]. Burnap et al. applied three feature learning methods including principal component analysis, low rank and sparse matrix decomposition, and exponential sparse restricted Boltzmann machine for quantitative preference model construction to predict customer choices [19]. Shi et al. developed a data-driven text mining and semantic network analysis approach based on probability and velocity correlation degree for design information retrieval [20]. Ma et al. proposed a systematic decision-making method for product development to evaluate function solution principles precisely based on fuzzy morphological matrix by using the information from customer preferences, product failures, and engineers’ knowledge [21].

On the other hand, with the increasing product complexity, manufacturers are paying more and more attention to ensure whether the desired performance is achieved within given design constraints when conducting product development [22,23]. It is always difficult for designers to analyze and validate product performance efficiently and effectively due to the limited professional knowledge and black-box models (a black-box model is an unknown function description that is given a list of design variables, and corresponding performance outputs can be acquired without knowing its expression). To ease this problem, performance analysis has become a hot topic for both academics and practitioners. According to the different phases of product development, the existing related works mainly can be classified into the following two types: the first one is conceptual design-centric, investigating the estimation of product performance under uncertain conditions in the early stage of design. Kalay proposed a performance-based design paradigm and defined performance as a measure of the expectation of the confluence form and function within a given context [24]. Coulibaly et al. presented a methodology to provide indicators for performance prediction at early stage of design by using computer-aided design model and an associated semantic matrix [25]. In order to obtain more accurate design results, Li et al. developed an effective strategies to resolve the performance coupled design problems based on performance model transformation, and proposed a qualitative analysis-based selection approach to handle the potential performance coupling [26]. Sun et al. put forward a behavioral design approach to help designers optimize product performance in the design phase by taking into account use conditions and requirements [27]. The other one focuses on detailed design-centric, exploring the efficient approaches to use approximation model to make performance analysis process more quickly and accurately. Approximation model can filter numerical noise and render a view of the entire design space, and then it is easy to detect errors in detailed design process. Leary et al. proposed a knowledge-based Kriging model for expensive function evaluation [28]. To reduce the computational expense, Zheng et al. developed an improved metamodeling approach based prior-knowledge and LSSVR to gain an accurate approximation for performance analysis and optimization [29]. Chen et al. proposed a simulation-based design framework based on geometric variability assessment and design-space dimensionality reduction by Karhunen–Loève expansion, metamodel and deterministic particle swarm optimization to realize shape performance optimization [30]. In order to make a trade-off between high accuracy and low expense, Zhou et al. presented an active learning variable-fidelity metamodeling approach based on model fusion and sequential sampling [31].

Although the above studies have made great contributions to performance analysis and product development, there is still lack of a systematical performance analysis approach in the early product development for industrial IoT. Firstly, how to explore the gathered data from implementing industrial IoT to support product development is rarely discussed. The huge product lifecycle data can provide tremendous value for product developers to exploit the implicit user preference. Especially, previous researches mainly focus on the detailed design process, and these have very limited means to support computational and dependable performance analysis process for conceptual design phase. Secondly, in order to deal with the complexity of performance analysis, extracting key design variables is a considerable issue for model simplification in data-driven product development. Thirdly, the uncertainty is a crucial factor to have a big influence on the accuracy of the forecast results, which is mainly from information loss and external disturbances. The approximation model is established to facilitate the performance analysis, and the information loss is inevitable to acquire the optimal model. Furthermore, due to the external disturbances of the collecting device, the uncertainty of the data samples is also considered. Therefore, in order to validate the design solution precisely, the uncertain factors such as data, model and result should be taken into account comprehensively. If some important performance parameters cannot be determined precisely, it will lead to extend product development time and increase costs.

To overcome the limitations of prior approaches, this paper develops a service-oriented layered architecture of industrial IoT for data-driven product development. Through the collected data from industrial IoT, a robust predicted performance analysis approach is proposed. The proposed approach is mainly divided into the following stages: the dimension reduction based on MI and outlier detection, the performance prediction based on LSSVR, and the performance analysis based on confidence interval. An empirical example is also shown to illustrate the applicability of the proposed approach. The main contribution of this paper is to develop a systematic and robust performance analysis framework to assist developers forecast product performance parameters in industrial IoT environment.

This paper is organized as follows: The detailed service-oriented layered architecture of industrial IoT for product development is presented in Section 2. The dimension reduction approach based on MI and outlier detection is proposed in Section 3. The performance prediction process based on LSSVR is put forward in Section 4. Section 5 proposes a performance analysis method based on confidence interval estimation to improve the robustness of the forecasting results. A case study about performance analysis of hydraulic press is provided in Section 6 to verify the effectiveness of the proposed approach. Finally, Section 7 concludes this paper and discusses about the future research plans.

## 2. Problem Description for Performance Analysis in Industrial IoT

### 2.1. Service-Oriented Layered Architecture of Industrial IoT

With the popularization and application of sensor network and wireless communication, industrial IoT provides intelligent information processing and smart control abilities to help manufacturing factories organize and manage lots of production systems effectively and efficiently. Analyzing the multidimensional dynamic data collected from different kinds of plant equipment, manufacturers can enable to conduct health monitoring, performance prediction, and remote diagnosis in real time to make better decisions for the optimization of production processes [32].

**Figure 1:**
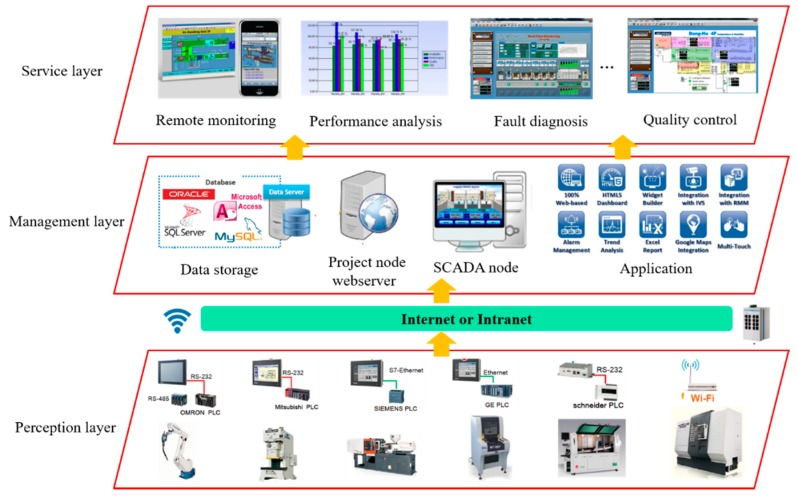
The service-oriented layered architecture of industrial IoT.

Figure 1 presents the service-oriented layered architecture of industrial IoT, which is applied to provide various applications by perceiving and processing product data. General speaking, the proposed architecture of industrial IoT is divided into the following layers: perception layer, management layer, and service layer: 

(1) Perception layer: this layer links the physical devices of factories though sensors, RFID readers and other data collection terminals [33,34] and the real-time state of manufacturing resources can be identified continuously. The perception interface is responsible for the collaboration and integration of all kinds of environments of sensor data; 

(2) Management layer: this layer is viewed as the brain of industrial IoT, which is introduced to manage a variety of heterogeneous data collected from perception layer. The management platform can assist manufacturers to make best-fit decisions by making use of these abundance data and extracting the helpful information; 

(3) Service layer: this layer is the entry of industrial IoT for manufactures, which is used to provide various high-quality services such as remote monitoring, performance analysis and fault diagnosis. The integrated service applications can be published in the form of micro-service, and then the manufactures can employ them on-demand as pay-as-you-go mode.

### 2.2. Performance Analysis for Data-Driven Product Development

**Figure 2:**
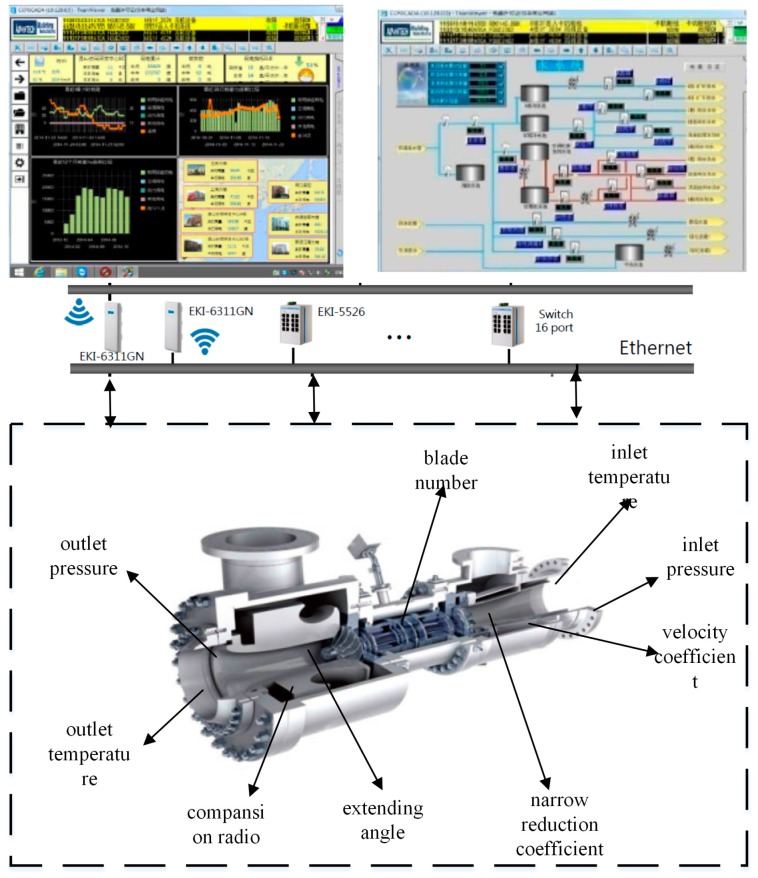
The illustration of the performance monitoring of turbo-expander in industrial IoT.

In general, the excellent performance depends on the reasonable defined ranges of the corresponding design parameters under the specific conditions. There are lots of industrial applications that are required to analyze the relationship between the performance and design parameters based on the collected data. Taking the product development of a turbo-expander for example, thermal conversion efficiency is very important performance index for developers to design and optimize in the product development. As a rule of thumb, there are about nine key design parameters such as inlet temperature, inlet pressure, and velocity coefficient that should be considered, which are closely related to the performance measurement. If the inlet pressure and inlet temperature are set out of scope, the thermal conversion efficiency will be observably reduced. Figure 2 shows the illustration of the performance monitoring of turbo-expander in industrial IoT. The factory accumulates the operating data for product service and developers can apply this data to provide a guidance to analyze the performance.

Due to the lack of an accurate mathematical description of the specific performance in the product development, developers tend to use approximation models to analyze and predict the performance for judging which solution is the best choice. In the product development process of industrial IoT, the performance analysis process of mechanical products mainly depends on the collected data from actual operation, simulation analysis and prior information. As a result, there are three important issues that should be considered in detail for the robust performance analysis.

Firstly, the establishment of the approximation model relies on the input variables which are defined and represented by developers. In order to reduce the computational complexity, it is necessary to select the performance-related variables as the inputs for the performance analysis. Secondly, it is necessary to take into account the efficient approaches for the construction of the approximation model that can render a view of the entire design space. The last but not the least, due to the ubiquity of uncertain information, it is always found that there is a large error between the estimated value and real value, so the performance analysis approach should consider the uncertainty of complex system and provide product developers dependable results to anticipate design defect accurately.

**Figure 3:**
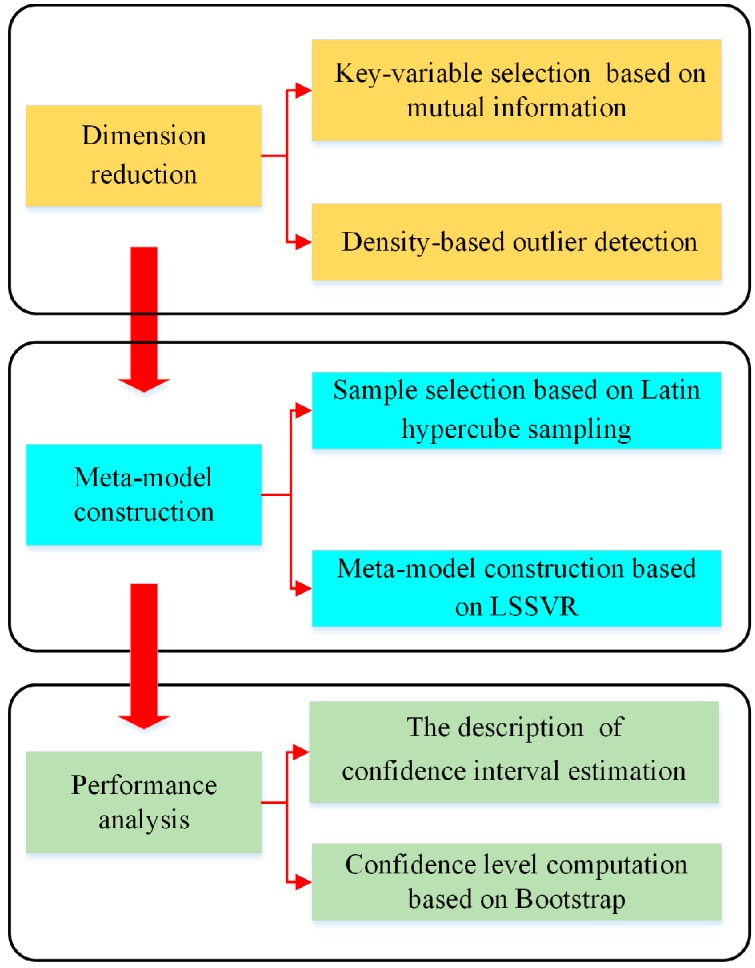
The overview of the robust performance analysis in product development.

Based on the above description, this paper puts forward a robust performance analysis approach to assist developers forecast and evaluate the key performance parameters in the early product development, as shown in Figure 3. According to the gathered data, the corresponding design variables are selected as the inputs and the LSSVR approach is introduced to establish the performance prediction model. Finally, the confidence interval estimation is incorporated into the forecasting process to deal with the uncertainty of the performance analysis.

## 3. The Dimension Reduction for Multivariate Design Variable

In order to enhance the effectiveness of performance analysis process, this section focuses on the robustness processing of the data samples, which mainly includes the following two steps: key-variable selection for dimension reduction and outlier detection for the improvement of model precision.

### 3.1. Key-Variable Selection Based on MI

Due to lots of the coupling design variables in data-driven product development process, it is always difficult for designers to create a reliable predictor to analyze product performance efficiently and accurately. Furthermore, the model constructed by all design variables will generate the curse of dimensionality and the computation time is also increased exponentially. So key-variable selection plays an important role in performance analysis which can identify and retain the important input variables whereas remove less important ones in the problems of interest so that the complexity of the design problems is reduced [35]. Many promising methods such as sensitivity analysis, MI and principle component analysis have been widely used to judge and measure the importance of variables. This paper introduces MI to select the key design variables because of the two advantages: (1) MI can measure different types of interaction including nonlinear ones; (2) It is robust to the noisy features [36].

MI is used to measure the dependent degree of two given design variables, which can be perceived as evaluating the information shared by the two given design variables. If the MI between two given variables is large (small), it represents two variables are closely (not closely) related. To guarantee the effect and accuracy of computation, the MI is estimated based on *k*-nearest neighbor distances which is first proposed by Kraskov [37].

Considering the design space Z=(X,Y), given two random design variables X and Y with joint pdf $p_{X,Y}(x,y)$, and marginal pdfs $p_{X}(x)$ and $p_{Y}(y)$, X is the set of input design variables, Y is the set of output responses, defining ε(i)/2 is the distances from $\left(x_{i},y_{i}\right)$ to its *k*-th neighbor, $\varepsilon_{x}(i)/2$ and $\varepsilon_{y}(i)/2$ mean the distances between the same points projected into the X, Y subspace, $n_{x}(i)$ and $n_{y}(i)$ mean the number of points $x_{j}$ and $y_{j}$, $\left\|x_{i}-x_{j}\right\|\leq \varepsilon_{x}(i)/2$, $\left\|y_{i}-y_{j}\right\|\leq \varepsilon_{y}(i)/2$. Then the estimation of MI can be developed as:

(1) $$\hat{I}(X;Y)=\psi (k)-\frac{1}{k}-\frac{1}{n}\sum_{i=1}^{n}\left[\psi (n_{x}(i)\right)+\psi (n_{y}(i))]+\psi (n)$$

where ψ(·) is the digamma function, $\psi (x)=\Gamma {\left(x\right)}^{-1}d\Gamma (x)/dx$, ψ(x+1)=ψ(x)+1/x, ψ(1)=−C, C=0.5772156649. The $I(X_{i};Y)$ between design variables $X_{i}$ and output response Y can be computed by Equation (1), and the selected threshold $\delta_{1}\in [0,1]$ is determined by the cross validation. Then the design variables which have $I(X_{i};Y)\geq \delta_{1}$ will be selected as the set of key design variables.

### 3.2. Density-Based Outlier Detection Approach for Data Model

Immense amounts of product data have been gathered from product lifecycles, which can provide a rich source of knowledge for product development, manufacturing and maintenance. Due to various factors such as equipment fault, human error and operating condition change, the collected product data is inevitably corrupted. The outlier which does not comply with the general behavior of the data model is viewed as careless error and should be rejected [38,39]. Appropriate data model has an important influence on the effective of the following meta-model construction, so the density-based outlier detection approach is applied to improve the veracity and reliability of the product data model.

Given a data set D, an object O and a positive integer *k*, $dist_{k}(O)$ is the neighbor radius of O, $dist(O,O^{\prime })$ represents the distance between object O and $O^{\prime }$, the *k*-distance neighborhood of O can be defined as $N_{k}(O)=\{O^{\prime }|O^{\prime }\in D,dist(O,O^{\prime })\leq dist_{k}(O))\}$. Then the local reachability density and local outlier factor of object O can be defined as Equations (2) and (3):

(2) $$lrd_{k}(O)=\frac{\left\|N_{k}(O)\right\|}{\sum_{O^{\prime }\in N_{k}(O)}\max\{dist_{k}(O),dist(O,O^{\prime })\}}$$

(3) $$LOF_{k}(O)=\frac{\sum_{O^{\prime }\in N_{k}(O)}\frac{lrd_{k}(O^{\prime })}{lrd_{k}(O)}}{\left\|N_{k}(O)\right\|}$$

**Figure 4:**
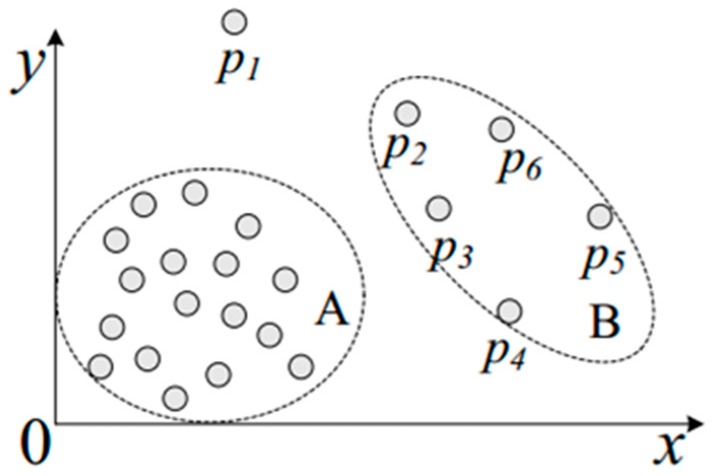
The illustration of the density-based outlier.

Local outlier factor means the average of the ratio of the reachability density of O and its *k*-nearest neighbors, and it can identify the point being an outlier, as shown in Figure 4. In the data-driven product development process, the outlier detection can determine the careless error from data model and it will improve the effectiveness and reliability of data preprocessing.

## 4. Meta-Model Construction and Performance Prediction Based on LSSVR

In the early product development, the performance prediction process can assist designers quickly understand whether the key performance features satisfy the design requirements, and this will reduce the probability of poor design results and improve design efficiency. Due to the lack of exact mathematical models, developers always feel powerless to obtain the qualitative performance estimation in specific working conditions. In response, this section introduces LSSVR-based approximation model to construct the response function between design variables and performance outputs for performance analysis.

### 4.1. Sample Selection Based on Latin Hypercube Sampling

The appropriate data sample is a vital factor to acquire available performance model for product development, which can use the fewer sample points to reflect the global design problem. Latin hypercube sampling (LHS) is an effective and powerful sampling approach for performing computer experiments [40], which partitions the n samples into m equally spaced intervals. In order to achieve uniform sampling for sample selection, an optimization criterion is defined as follows:

(4) $$\max\{\min d(x_{i},x_{j})\}$$

where $d(x_{i},x_{j})=\sqrt{\left[\sum_{k=1}^{m}{\left|x_{ik}-x_{jk}\right|}^{2}\right]}$, and $d(x_{i},x_{j})$ represents the distance between sample points. Applying the optimization criterion can obtain the samples which have the better uniformity and filling ability, and make all samples have a balanced distribution in the design space. Furthermore, Z-score is used to normalize the data sample to eliminate dimension so that data have the same caliber. Suppose there is a sampled data set $X\in R^{m\times f}$, f is the number of design variables, m is the number of the sample, so the classic Z-score can be defined as:

(5) $$z_{i}=\frac{x_{i}-m(X)}{s(X)}$$

where $x_{i}$ is the sample of data set X, m(X) is the mean of the all data of sampled data set, s(X) is the corresponding standard deviation.

### 4.2. Meta-Model Construction Based on Least Squares Support Vector Regression

Due to the time-consuming and computation-prohibitive performance analysis process, building a metamodel is considered as a promising approach to describe the implicit approximate relationship between design variables and product performance. Support vector regression (SVR) is one of the most popular meta-models with easy generation and low standard deviation [41], which can fit a linear function of the approximate relationship with least reasonable complexity by learning and training the original data.

The least squares version of SVR, named LSSVR, has gained lots of attention because of its low complexity and low computational cost. LSSVR only requires to solve a linear equation as a surrogate of quadratic programming so that it can reduce the training complexity obviously. Given a set of standardized training data ${\left\{x_{i},y_{i}\right\}}_{i=1}^{m}$, $x_{i}$ ($x_{i}\in R^{m}$) is the input variable, and $y_{i}$ ($y_{i}\in R^{m}$) is the output data, then the LSSVR is formulated as follows [42]:

(6) $$\begin{array}{l} \min\Phi (w)=\frac{1}{2}\omega^{T}\omega +\frac{1}{2}C\sum_{i=1}^{m}\varepsilon_{i}^{2}\\ s.t.\;y_{i}=\omega^{T}\varphi (x_{i})+b+\varepsilon_{i},\;i=1,2,\ldots ,m \end{array}$$

where $\varphi :R^{m}\to R^{n}$ is a mapping function that maps the input data into a higher dimensional feature space, C is the regularization parameter, $\varepsilon_{i}$ represents error variance, ω is a weight vector and b is a bias term. So the corresponding Lagrange function can be defined as:

(7) $$L(\omega ,\xi ,\xi^{*},\alpha ,\alpha^{*})=\frac{1}{2}\omega^{T}\omega +\frac{1}{2}C\sum_{i=1}^{m}\varepsilon_{i}^{2}-\sum_{i=1}^{m}\alpha_{i}[\omega^{T}\varphi (x_{i})+b+\varepsilon_{i}-y_{i}]$$

Though applying Lagrange multipliers and taking the Karush-Kuhn-Tucker conditions for optimality, the least squares-based estimation function is formulated as:

(8) $$\hat{f}(x)=\sum_{i=1}^{l}\alpha_{i}K(x_{i},x)+b$$

where $\alpha ={\left(H+E/C\right)}^{-1}(Y-Ib)$, $b=\frac{I^{\prime }{\left(H+E/C\right)}^{-1}Y}{I^{\prime }{\left(H+E/C\right)}^{-1}I}$, $I={\left[1,1,\cdots ,1\right]}^{T}$, $Y=\left[y_{1},y_{2},\cdots ,y_{l}\right]$, E is the unit matrix of l×l, $H(i,j)=K(x_{i},x_{j})$, K(·,·) means the selected kernel function.

To reduce the computational cost, this paper uses the radial basis function (RBF) kernel as the kernel function of meta-model, and the RBF kernel can be presented as:

(9) $$K(x_{i},x)=\exp(-{\left\|x-x_{i}\right\|}^{2})/2\sigma^{2}$$

where σ is the depth of the RBF. In order to enhance the prediction accuracy, the kernel parameter σ and the regularization parameter C should be optimized to obtain the higher prediction performance. The performance index of meta-model can be defined by the mean squared error, and the mathematical model for the following optimization can be expressed as Equation (10):

(10) $$\begin{array}{l} \min f=\frac{1}{m}\sum_{i=1}^{m}{\left(y_{i}-{\hat{y}}_{i}\right)}^{2}\\ s.t.\;C\in [C_{\min},C_{\max}],\;\sigma \in [\sigma_{\min},\sigma_{\max}] \end{array}$$

To date, many parameter optimization methods such as cross validation, theoretical analysis and heuristic algorithm have been developed by experts and scholars. This paper introduces particle swarm optimization (PSO) algorithm to obtain the optimal solution for the meta-model construction of performance prediction. PSO is a population-based stochastic optimization technique proposed by Kennedy et al., inspired by the social behavior of bird foraging [43]. This approach can search a best location which has the best fitness values for objective functions by giving a direction of particles. Compared to the meta heuristics such as genetic algorithm and strength Pareto evolutionary algorithm, PSO has some advantages such as simple mathematical operators and high efficient running speed. This section regards performance index as fitness function, and applies PSO to seek the best solution as the optimal parameters.

## 5. Predicted Performance Analysis Based on Confidence Interval Estimation

The predicted performance results are always uncertain due to the various uncertain factors such as model uncertainty, information loss and external disturbances. It is difficult to describe the accurate and comprehensive performance using real number representation. Product developers will adjust design variables considering the worst case scenario in the absence of confidence intervals, and this will increase the complexity and unreliability of product development. So this section extends the predicted performance using confidence interval estimation to improve the robustness of performance analysis.

To improve the effectiveness of predicted results, interval estimation is a popular tool to cope with the influence about model uncertainty and data uncertainty [44]. Bootstrap is an effective resampling approach which is applied to determine confidence intervals on a quantity of interest using only one design of experiments [45]. It is well suited for population distribution to statistical inference without the distribution hypothesis. Suppose the sample error is independent identically distributed, $y_{i}$ is the *i*-th true value of the performance response, ${\hat{y}}_{i}$ is the *i*-th predicted performance. Sample randomly *N* times from the data samples, repeat *L* times, and then obtain a training sample set ${\left\{D_{il}\right\}}_{l=1}^{L}$. Though the performance prediction process mentioned above, the corresponding predicted performance can be represented as ${\left\{{\hat{y}}_{i}\right\}}_{l=1}^{L}$. So the finally average of performance prediction ${\bar{\hat{y}}}_{i}$, the prediction variance $\sigma_{{\hat{y}}_{i}}^{2}$, and the variance of the data noise $\sigma_{{\hat{\varepsilon }}_{i}}^{2}$ by bootstrap can be formulated as:

(11) $$\left\{ \begin{array}{l} {\bar{\hat{y}}}_{i}=\frac{1}{L}\sum_{l=1}^{L}{\hat{y}}_{i}\\ \sigma_{{\hat{y}}_{i}}^{2}=\frac{1}{L-1}\sum_{l=1}^{L}{\left(y_{i}-{\hat{y}}_{i}\right)}^{2}\\ \sigma_{{\hat{\varepsilon }}_{i}}^{2}=E\{{\left(y^{*}-\hat{y}\right)}^{2}\}-\sigma_{{\hat{y}}_{i}}^{2} \end{array}\right.$$

**Figure 5:**
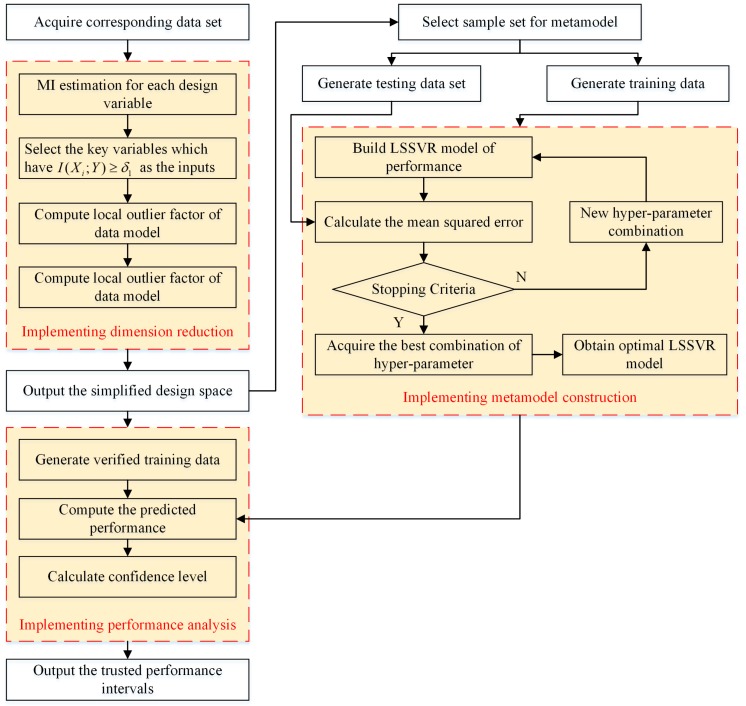
The flowchart of the proposed performance analysis approach.

Then the approximate 100(1−α)% pointwise (in point *x*) confidence intervals can take the form:

(12) $$PI_{i}=({\hat{y}}_{i}-t_{df}^{1-\frac{\alpha }{2}}\sqrt{\sigma_{{\hat{y}}_{i}}^{2}+\sigma_{{\hat{\varepsilon }}_{i}}^{2}},{\hat{y}}_{i}+t_{df}^{1-\frac{\alpha }{2}}\sqrt{\sigma_{{\hat{y}}_{i}}^{2}+\sigma_{{\hat{\varepsilon }}_{i}}^{2}})$$

where $t_{df}^{1-\frac{\alpha }{2}}$ denotes the ($1-\frac{\alpha }{2}$)th quantile of the standard *t* distribution. To summarize, Figure 5 provides an illustration of the flowchart of the proposed performance analysis approach.

## 6. Case Study

**Figure 6:**
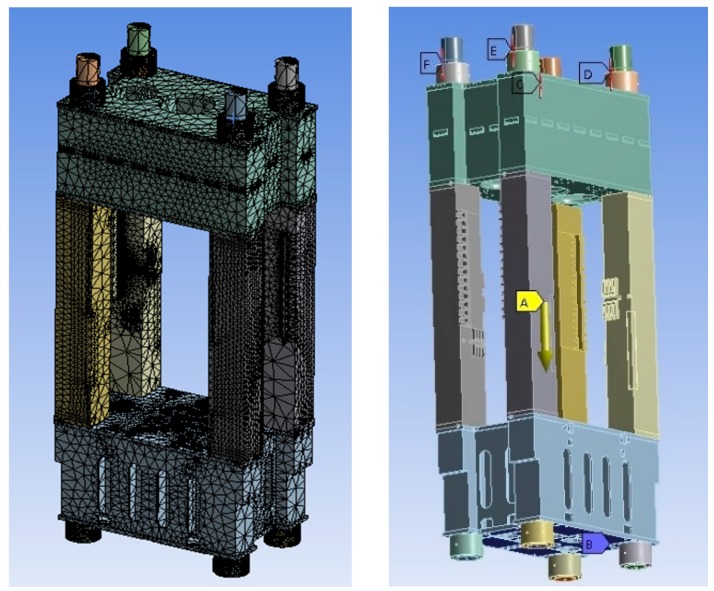
The simulation model of a hydraulic press.

Due to the huge mass and bearing size of heavy-duty hydraulic presses, they always use a composite frame style to conduct product development. Under the condition of mutation overloading, the bends of the composite frame structure will appear high local stress area to result in the unrepairable fatigue crack. In response, the prestressed composite structure is considered as the popular way in product development of the bearing component. Developers define the overall performance to describe the situation that the adjoining planes of the composite structure don’t appear fatigue crack in the case of loading and unloading. The overall performance has a major impact on product quality in the engineering design of hydraulic press. The slotted coefficient and gap length of the adjoining planes are always used to evaluate the overall performance at present. In order to validate the effectiveness of the proposed paper, this paper selects the slotted coefficient as the evaluation indicator. Figure 6 presents the simulation model of hydraulic press.

Traditional overall performance analysis of the hydraulic press mainly depends on the simulation and verification of the physical prototype, and it will greatly increase the development costs. In the industrial IoT environment, the product lifecycle data which is from product development, manufacturing and service process can be collected by networks, and developers are convenient to acquire this valuable data to produce high-quality products. 

**Figure 7:**
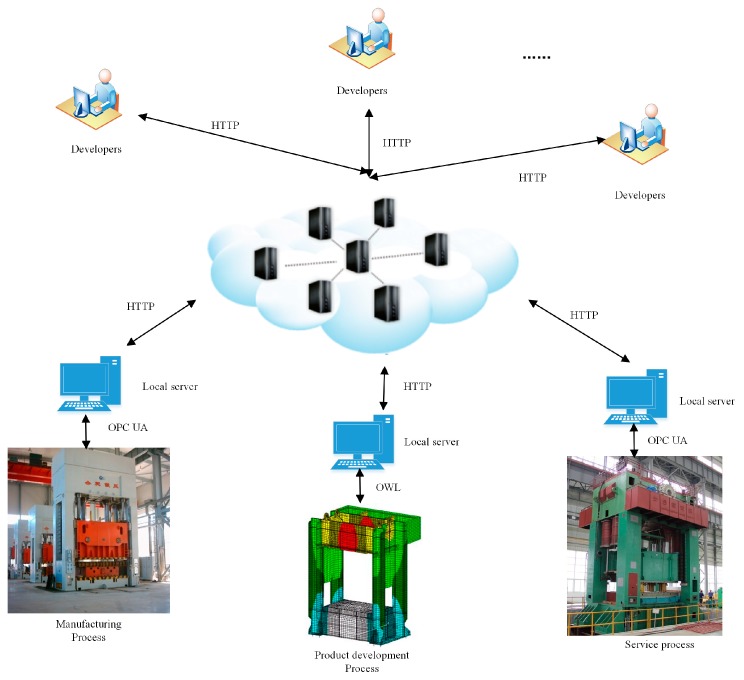
The framework of the data gathering of hydraulic press in industrial IoT.

Figure 7 presents the framework of the data gathering of hydraulic press in industrial IoT. According to enterprise practical situations, this paper uses the historical data collected from experimental simulation and service process as the data sources for the overall performance analysis. To improve the effectiveness of the data model, data preprocessing is first conducted. Due to the complex design conditions, this paper takes 200 samples as example, and Table 1 shows the specific design variables of the partial samples. Moreover, these design variables are supposed to be normally distributed.

In general, the design variables mentioned in Table 1 above have different influences on the overall performance. For example, some variables may have a strong impact on the performance of the horizontal beam, however, these may have little impact on the overall performance. To simplify the product development process, MI is used to assess the relationship between the overall performance and design variables, and Table 2 shows the value of MI between the design variables and the overall performance. Conducting cross-validation process with the objective function of minimizing the mean value of overall performance error, and determining the threshold $\delta_{1}=2.5$. So the important variables of the overall performance are selected as $I(X_{i};Y)\geq 2.5$, and the detailed design variables is selected as follows: pretightening force, tie rod diameter, eccentricity, tie rod bias, stiffness ratio, cross-sectional area of stand column, and relative deflection. Furthermore, the outlier detection approach is used to eliminate the wrong samples and there are 160 remaining samples to conduct the following performance analysis.

**Figure 8:**
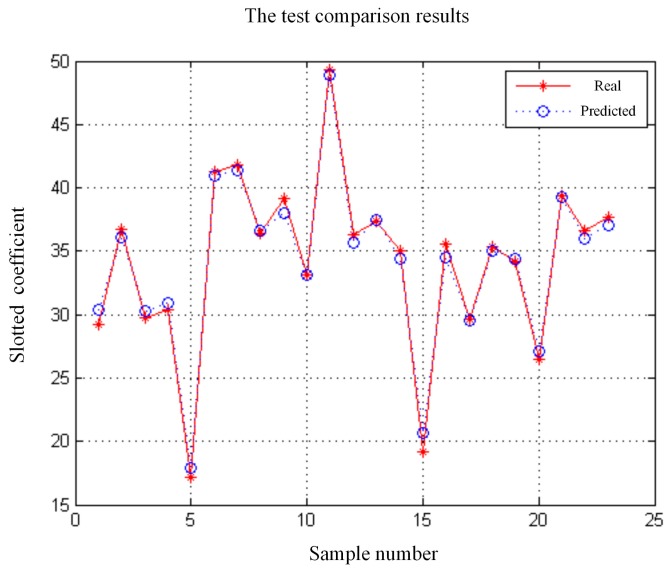
The test comparison results.

The performance prediction process applies the 137 samples as the training sample, and the rest samples as the test sample. The program of LSSVR algorithm is programmed in Matlab 2010b environment and run on a desktop computer equipped with a dual 2.63 GHz Intel i5 processor and 8 GB RAM. In order to obtain the optimal prediction model, the kernel parameter σ and the regularization parameter C should be determined by applying PSO. Through the iterative optimization, the optimal parameters of the LSSVR can be acquired as σ=3.744, C=42.533. Figure 8 shows the test comparison results of the proposed performance prediction model. The mean square error (MSE) and the R square value (R^2^) are introduced as the accuracy metrics to verify the feasibility of the approximation model. According to verify the test sample, the testing performance (MSE, R^2^) of the approximation model is (0.003, 0.9806). The lower value of MSE, and the larger value of R square, the more accurate the metamodel.

According to the working conditions of the factory, there are four design solutions of the press should be considered as alternatives for users. Applying the established metamodel to predict the overall performance, and the slotted coefficient can be obtained as S_1_ = 2.7693, S_2_ = 1.6971, S_3_ = 3.7346, and S_4_ = 2.1549. In order to analysis the confidence interval of the predicted results, bootstrap is used to resampling 1000 times to acquire the bootstrap sequence of the slotted coefficient. Table 3 represents the confidence interval estimation of the partial prediction results with the 0.95 confidence coefficient. Based on the performance analysis process, it can find that stiffness ratio is the most important design factor for the overall performance. It is consistent with the actual application, and in order to improve the overall performance, the design rules should be adopted with the large tie rod diameter, the small cross-sectional area of stand column, the large relative deflection and the big tie rod bias.

To investigate further the superiority of the proposed approach in performance analysis in the early product development, a fair comparison is made among the standard SVR with MI, Kriging model with MI and ANN with MI. The results are represented in Table 4. It shows that the proposed approach outperforms the standard SVR, Kriging model and ANN both prediction accuracy and computing time. Furthermore, confidence interval is used to provide the performance description by the proposed approach, and this manner can take into account the uncertainty to support more robust representation for developers’ decision-making. The result can serve as a reference for performance estimation in product development.

## 7. Conclusions

With the high-speed development of information and communication technology, industrial IoT is becoming a popular intelligent manufacturing mode to manage and optimize the entire product manufacturing process in real time. Manufacturers can implement operation management, real-time monitoring and performance prediction to ensure the stable operation of the production platform. To improve the product quality in the early product development, a robust performance analysis approach in industrial IoT environment is proposed. The detailed service-oriented layered architecture of industrial IoT for data-driven product development is developed to describe the relationship between industrial IoT and product development. In the face of the high dimensional design space, the dimension reduction approach based on MI and outlier detection is put forward. And then a performance prediction approach based on LSSVR is applied to explore the implicit performance meta-model. Furthermore, a confidence interval-based predicted performance analysis considering the uncertainty is adopted to improve the robustness of the forecasting results. In the future, we will plan to explore the more effective intelligent algorithms considering the big data environment to improve the practicality of the proposed approach.

## Figures and Tables

**Table 1:** The detailed description of design variables.

No	Design Variables	Sample 1	Sample 2	Sample 3	Sample 4	Sample 5
X_1_	Tie rod diameter (mm)	670	704	735	765	794
X_2_	Tie rod bias (mm)	0	10	20	30	40
X_3_	Cross-sectional area of stand column (mm^2^)	848,583	707,152	606,131	530,364	471,435
X_4_	Flexural coefficient	16.12	19.37	25.99	29.82	22.68
X_5_	Pretightening force (MN)	11.63	13.09	14.55	16.00	17.46
X_6_	Stiffness ratio	0.3319	0.3663	0.3994	0.4327	0.4661
X_7_	Moment of inertia (cm^4^)	1.6 × 108	1.45 × 108	1.57 × 108	1.49 × 108	1.5 × 108
X_8_	Relative deflection (mm/m)	0.22	0.25	0.31	0.28	0.30
X_9_	Eccentricity (mm)	0	100	200	300	400
X_10_	Inside gap (mm)	0.5	1.0	2.0	0	1.5
X_11_	Swaging (mm)	9.1	5.48	0.45	0.56	0
X_12_	Saddle forging (mm)	28.45	23.67	0	15.71	0
X_13_	Eccentric heading (mm)	23.56	14.67	15.32	0	0
X_14_	Surface pressure (MPa)	13.3	15.6	14.5	16.1	13.9

**Table 2:** The value of MI between the design variables and the overall performance.

No	Overall Performance	No	Overall Performance
X_1_	2.7	X_8_	2.9
X_2_	3.5	X_9_	2.6
X_3_	3.2	X_10_	2.1
X_4_	1.3	X_11_	1.9
X_5_	2.8	X_12_	1.8
X_6_	3.6	X_13_	1.2
X_7_	1.5	X_14_	1.7

**Table 3:** The confidence interval estimation.

No	The Predicted Performance	Confidence Upper Limit	Confidence Lower Limit	Confidence Coefficient
S_1_	2.7693	2.5308	3.1077	0.95
S_2_	1.6971	1.3574	1.9875	0.95
S_3_	3.7346	3.2145	4.1047	0.95
S_4_	2.1549	1.8546	2.5674	0.95

**Table 4:** Comparison between the proposed approach and the other approaches using the same experimental data.

Prediction Model	MAPE (%)	Computing Time (s)	Performance Description
MI + SVR	2.5489	25.87	real number
MI + Kriging	1.6784	20.21	real number
MI + ANN	1.4895	24.69	real number
Proposed approach	0.8579	18.62	interval
